# Supplementary material for: Human Milk Antibodies against S1 and S2 Subunits from SARS-CoV-2, HCoV-OC43, and HCoV-229E in Mothers with a Confirmed COVID-19 PCR, Viral SYMPTOMS, and Unexposed Mothers
Source: Int J Mol Sci. 2021 Feb 9;22(4):1749. doi: 10.3390/ijms22041749 (PMC7916441; doi:10.3390/ijms22041749)
Supplement: Supplementary file 1 [file ijms-22-01749-s001.pdf]

Table S1. Participant characteristics[1] with a confirmed COVID-19 PCR test (M1 to M7). The control group (C1 to C6) represents unexposed mothers who donated human milk samples before the COVID-19 pandemic in 2018.

Mothers	Confirmed COVID-19 PCR (Nasal Swab)	COVID-19 Symptoms	Time from Infection to Collection (Days)	Infant Gender	Lactation Time (Months)	Maternal Age
**M1**	+COVID19 PCR	Fever, fatigue, cough	61	Female	5	36
**M2**	+COVID-19 PCR	Cough, fatigue, fever, loss of taste/smell	60	Female	6	26
**M3**	+RNA SARS-CoV-2 PCR	Fever, nasal congestion, and cough	84	Female	6	26
**M4**	+COVID-19 PCR	Fatigue, fever	40	Male	6	33
**M5**	+COVID-19 PCR	Fever and cough	16	Male	8	37
**M6**	+RNA SARS-CoV-2 PCR	Cough and fever	47	Male	8	31
**M7**	+COVID-19-PCR	Headaches, fatigue, fever	18	Male	5	33
**C1**	-	Unknown	-	Male	8	28
**C2**	-	Unknown	-	Female	5	37
**C3**	-	Unknown	-	Male	7	29
**C4**	-	Unknown	-	Female	5	35
**C5**	-	Unknown	-	Male	5	33
**C6**	-	Unknown	-	Female	7	27

[1] Women did not have a medical history, medication and systematic diseases.

A S1 subunit SARS-CoV-2
P0DTC2|13-685 (YP_009724390.1)
SQCVNLTTRTQLPPAYTNSFTRGVYYPDKVFRSSVLHSTQDLFLPFFSNVTWFHAIHVSG
TNGTKRFDNPVLPFNDGVYFASTEKSNIIRGWIFGTTLDSKTQSLLIVNNATNVVIKVCE
FQFCNDPFLGVYYHKNNKSWMESEFRVYSSANNCTFEYVSQPFLMDLEGKQGNFKNLREF
VFKNIDGYFKIYSKHTPINLVRDLPQGFSALEPLVDLPIGINITRFQTLLALHRSYLTPG
DSSSGWTAGAAAYYVGYLQPRTFLLKYNENGTITDAVDCALDPLSETKCTLKSFTVEKGI
YQTSNFRVQPTESIVRFPNITNLCPFGEVFNATRFASVYAWNRKRISNCVADYSVLYNSA
SFSTFKCYGVSPTKLNDLCFTNVYADSFVIRGDEVRQIAPGQTGKIADYNYKLPDDFTGC
VIAWNSNNLDSKVGGNYNYLYRLFRKSNLKPFERDISTEIYQAGSTPCNGVEGFNCYFPL
QSYGFQPTNGVGYQPYRVVVLSFELLHAPATVCGPKKSTNLVKNKCVNFNFNGLTGTGVL
TESNKKFLPFQQFGRDIADTTDAVRDPQTLEILDITPCSFGGVSVITPGTNTSNQVAVLY
QDVNCTEVPVAIHADQLTPTWRVYSTGSNVFQTRAGCLIGAEHVNNSYECDIPIGAGICA
SYQTQTNSPRRAR

B S2 subunit SARS-CoV-2
P0DTC2|686-1273 (YP_009724390.1)
SVASQSIIAYTMSLGAENSVAYSNNSIAIPTNFTISVTTEILPVSMTKTSVDCTMYICGD
STECSNLLLQYGSFCTQLNRALTGIAVEQDKNTQEVFAQVKQIYKTPPIKDFGGFNFSQI
LPDPSKPSKRSFIEDLLFNKVTLADAGFIKQYGDCLGDIAARDLICAQKFNGLTVLPPLL
TDEMIAQYTSALLAGTITSGWTFGAGAALQIPFAMQMAYRFNGIGVTQNVLYENQKLIAN
QFNSAIGKIQDSLSSTASALGKLQDVVNQNAQALNTLVKQLSSNFGAISSVLNDILSRLD
KVEAEVQIDRLITGRLQSLQTYVTQQLIRAAEIRASANLAATKMSECVLGQSKRVDFCGK
GYHLMSFPQSAPHGVVFLHVTYVPAQEKNFTTAPAICHDGKAHFPREGVFVSNGTHWFVT
QRNFYEPQIITTDNTFVSGNCDVVIGIVNNTVYDPLQPELDSFKEELDKYFKNHTSPDVD
LGDISGINASVVNIQKEIDRLNEVAKNLNESLIDLQELGKYEQYIKWPWYIWLGFIAGLI
AIVMVTIMLCCMTSCCSCLKGCCSCGSCCKFDEDDSEPVLKGVKLHY

C S1+S2 HCoV-229E
A0A1L7B942 (APT69883.1)
MFVLLVAYALLHIAGCQTTNGTNTSHSVCNGCVGHSENVFAVESGGYIPSNFSFNNWFLL
TNTSSVVDGVVRSFQPLLLNCLWSVSGSQFTTGFVYFNGTGRGACKGFYSNASSDVIRYN
INFEENLRRGTILFKTSYGAVVFYCTNNTLVSGDAHIPSGTVLGNFYCFVNTTIGNETTS
AFVGALPKTVREFVISRTGHFYINGYRYFSLGDVEAVNFNVTNAATIVCTVALASYADVL
VNVSQTAIANIIYCNSVINRLRCDQLSFDVPDGFYSTSPIQPVELPMSIVSLPVYHKHTF
IVLHVKFEHQRGPGKCYNCRPSVINITLANFNETKGPLCVDTSHFTTQFVDNVKLARWSA
SINTGNCPFSFGKVNNFVKFGSVCFSLKDIPGGCAMPIMANLVNHKSHNIGSLYVSWSDG
DVITGVPKPVEGVSSFMNVTLNKCTKYNIYDVSGVGVIRISNDTFLNGITYTSTSGNLLG
FKDVTNGTIYSITPCNPPDQLVVYQQAVVGAMLSENFTSYGFSNVVEMPKFFYASNGTYN
CTDAVLTYSSFGVCADGSIIAVQPRNVSYDSVSAIVTANLSIPSNWTTSVQVEYLQITSK
PIVVDCSTYVCNGNVRCVELLKQYTSACKTIEDALRNSAMLESADVSEMLTFDKKAFTLA
NVSSFGDYNLSSVIPSLPRSGSRVAGRSAIEDILFSKLVTSGLGTVDADYKKCTKGLSIA
DLACAQYYNGIMVLPGVADAERMAMYTGSLIGGIALGGLTSAASIPFSLAIQSRLNYVAL
QTDVLQENQKILAASFNKAMTNIVDAFTGVNDAITQTSQALQTVATALNKIQDVVNQQGN
SLNHLTSQLRQNFQAISSSIQAIYDRLDIIQADQQVDRLITGRLAALNVFVSHTLTKYTE
VRASRQLAQQKVNECVKSQSKRYGFCGNGTHIFSLVNAAPEGLVFLHTVLLPTQYKDVEA
WSGLCVDGINGYVLRQPNLALYKEGNYYRITSRIMFEPRIPTIADFVQIENCNVTFVNIS
RSELQTIVPEYIDVNKTLQELSYKLPNYTVPDLVVEQYNQTILNLTSEISTLENKSAELN
YTVQKLQTLIDNINSTLVDLKWLNRVETYIKWPWWVWLCISVVLIFVVSMLLLCCCSTGC
CGFFSCFASSIKGCCESTKLPYYDVEKIHIQ

D S1+S2 HCoV-OC43
A0A2P1ZWW8 (AVR40344.1)
MFLILLISLPTAFAVIGDLNCTLDPRLKGSFNNRDTGPPSISIDTVDVTNGLGTYYVLDR
VYLNTTLFLNGYYPTSGSTYRNMALKGTDLLSTLWFKPPFLSDFINGIFAKVKNTKVFKD
GVMYSEFPAITIGSTFVNTSYSVVVQPRTINSTQDGVNKLQGLLEVSVCQYNMCEYPHTI
CHPNLGNHFKELWHYDTGVVSCLYKRNFTYDVNATYLYFHFYQEGGTFYAYFTDTGFVTK
FLFNVYLGMALSHYYVMPLTCIRRPKDGFSLEYWVTPLTPRQYLLAFNQDGIIFNAVDCM
SDFMSEIKCKTQSIAPPTGVYELNGYTVQPVADVYRRKPDLPNCNIEAWLNDKSVPSPLN
WERKTFSNCNFNMSSLMSFIQADSFTCNNIDAAKIYGMCFSSITIDKFAIPNRRKVDLQL
GNLGYLQSSNYRIDTTATSCQLYYNLPAANVSVSRFNPSTWNKRFGFIEDSVFVPQPTGV
FTNHSVVYAQHCFKAPKNFCPCSSCSCPGKNNGIGTCPAGTNSLTCDNLCTLDPITLKAP
DTYKCPQSKSLVGIGEHCSGLAVKSDYCGNNSCTCQPQAFLGWSADSCLQGDKCNIFANF
ILHDVNNGLTCSTDLQKANTEIELGVCVNYDLYGISGQGIFVEVNATYYNSWQNLLYDSN
GNLYGFRDYITNRTFMIHSCYSGRVSAAYHANSSEPALLFRNIKCNYVFNNSLTRQLQPI
NYSFDSYLGCVVNAYNSTAISVQTCDLTVGSGYCVDYSKNRRSRRAITTGYRFTNFEPFT
VNSVNDSLEPVGGLYEIQIPSEFTIGNMEEFIQTSSPKVTIDCAAFVCGDYAACKLQLVE
YGSFCDNINAILTEVNELLDTTQLQVANSLMNGVTLSTKLKDGVNFNVDDINFSPVLGCL
GSECSKASSRSAIEDLLFDKVKLSDVGFVEAYNNCTGGAEIRDLICVQSYKGIKVLPPLL
SENQISGYTLAATSASLFPPWTAAAGVPFYLNVQYRINGLGVTMDVLSQNQKLIANAFNN
ALHAIQQGFDATNSALVKIQAVVNANAEALNNLLQQLSNRFGAISASLQEILSRLDALEA
EAQIDRLINGRLTALNAYVSQQLSDSTLVKFSAAQAMEKVNECVKSQSSRINFCGNGNHI
ISLVQNAPYGLYFIHFNYVPTKYVTAKVSPGLCIAGNRGIAPKSGYFVNVNNTWMYTGSG
YYYPEPITENNVVVMSTCAVNYTKAPYVMLNTSIPNLPDFKEELDQWFKNQTSVAPDLSL
DYINVTFLDLQVEMNRLQEAIKVLNHSYINLKDIGTYEYYVKWPWYVWLLICLAGVAMLV
LLFFICCCTGCGTSCFKKCGGCCDDYTGYQELVIKTSHDD

Figure S1. Amino acid sequences from (**A**) S1 subunit and (**B**) S2 subunit from SARS-CoV-2, S1 + S2 subunits from (**C**) HCoV-229E and (**D**) HCoV-OC43. Sequences were from UniProt (ELIXIR core data resource).

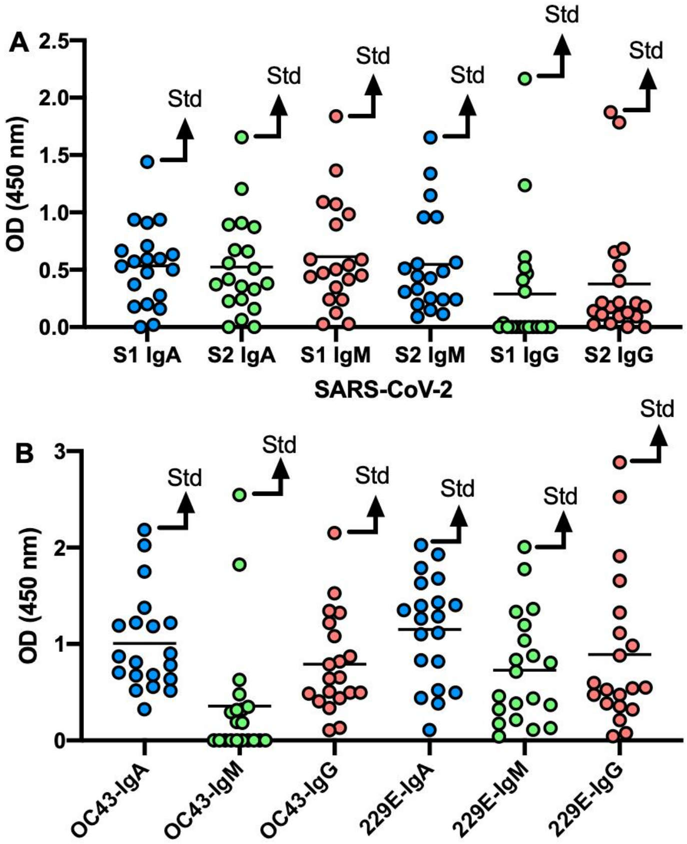

Figure S2. Selection of standards (std) with the highest optical density (OD) values using ELISA. The OD values for antibody secretory IgA (SIgA)/IgA, secretory IgM (SIgM)/IgM, and IgG reactive to (**A**) S1 or S2 subunit SARS-CoV-2, (**B**) S1+S2 subunits HCoV-OC43, or S1+S2 subunits HCoV-229E. The standards were selected among 20 human milk samples (supernatants) diluted at 10x for IgG and IgM and at 25x for SIgA/IgA in blocking buffer.

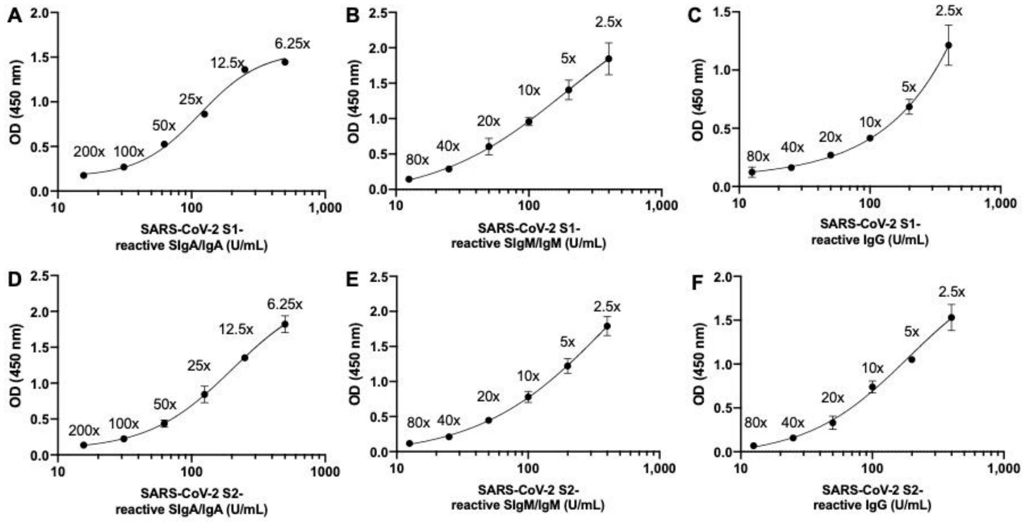

Figure S3. Standard curves used to determine the levels of antibodies reactive to SARS-CoV-2 S1 or S2 subunit. Standard curves for SARS-CoV-2 S1-reactive **a** secretory IgA (SIgA)/IgA, **b** secretory IgM (SIgM)/IgM, and **c** IgG. Standard curves for SARS-CoV-2 S2-reactive **d** SIgM/IgM, **e** SIgA/IgA, and **f** IgG. Values are means ± SD, n = 3 for experiments. The standard curves were prepared using the human milk supernatant with the highest optical density values (see Figure S2).

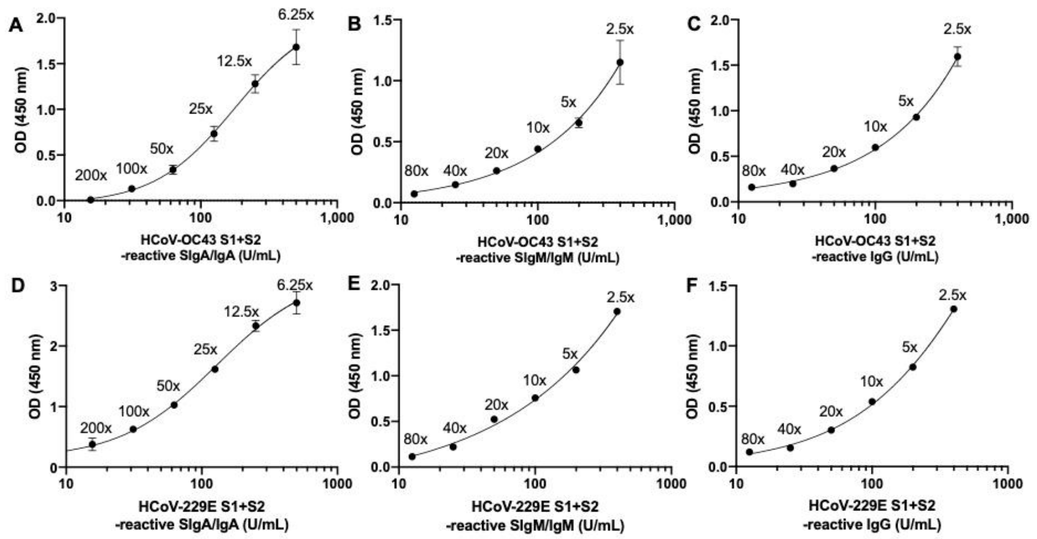

Figure S4. Standard curves used to determine the levels of antibodies reactive to HCoV-OC43 and HCoV-22E S1+S2 subunits. Standard curves for HCoV-OC43 S1+S2-reactive **a** secretory IgA (SIgA)/IgA, **b** secretory IgM (SIgM)/IgM, and **c** IgG. Values are means ± SD, n = 3 for experiments. The standards were prepared using the human milk supernatant with the highest optical density values (see Figure S2).
